# Two-Carbon Homologation of Aldehydes and Ketones to α,β-Unsaturated Aldehydes

**DOI:** 10.3390/molecules16065062

**Published:** 2011-06-17

**Authors:** Richard J. Petroski, Karl Vermillion, Allard A. Cossé

**Affiliations:** 1 Unit of Crop Bioprotection Research, USDA, Agricultural Research Service, National Center for Agricultural Utilization Research, 1815 N. University Street, Peoria, IL 61604, USA; 2 Unit of Functional Food Research, USDA, Agricultural Research Service, National Center for Agricultural Utilization Research, 1815 N. University Street, Peoria, IL 61604, USA

**Keywords:** aldehyde homologation, ketone to aldehyde homologation, diethyl methylformyl-phosphonate dimethylhydrazone, diethyl ethylformyl-2-phosphonate dimethylhydrazone

## Abstract

Phosphonate reagents were developed for the two-carbon homologation of aldehydes or ketones to unbranched- or methyl-branched α,β-unsaturated aldehydes. The phosphonate reagents, diethyl methylformyl-2-phosphonate dimethylhydrazone and diethyl ethylformyl-2-phosphonate dimethylhydrazone, contained a protected aldehyde group instead of the usual ester group. A homologation cycle entailed condensation of the reagent with the starting aldehyde, followed by removal of the dimethylhydrazone protective group with a biphasic mixture of 1 M HCl and petroleum ether. This robust two-step process worked with a variety of aldehydes and ketones. Overall isolated yields of unsaturated aldehyde products ranged from 71% to 86% after the condensation and deprotection steps.

## 1. Introduction

A recurring theme in the synthesis of various polyketide natural products and other compounds is two-carbon homologation of aldehydes or ketones to unbranched or branched (e.g., α-methyl or β-methyl) α,β-unsaturated aldehydes. A typical approach based on the Horner-Wadsworth-Emmons condensation [1] uses a phosphonate ester, such as triethyl-2-phosphonoacetate or triethyl-2-phosphonopropionate, and involves three synthetic steps: The phosphonate condensation step extends the chain by two carbons while introducing an (*E*) double bond, an α-alkyl branch (size depending on the choice of phosphonate reagent), and a terminal ester group. The ester is then reduced to an α,β-unsaturated alcohol, and the alcohol is partially oxidized to the desired α,β-unsaturated aldehyde. One way to shorten this scheme would be to use a homologating reagent that keeps the oxidation state of an aldehyde throughout.

A variety of options were considered. Aldehyde Wittig reagents, such as α-formylethylidenetriphenylphosphorane are known [2,3], but in our hands the reagent was not reactive toward α,β-branched unsaturated aldehydes, such as (2*E*)-2-methyl-2-butenal [4]. Phosphonate anions are generally more reactive toward aldehydes or ketones than the analogous phosporanes [5]. Aldehyde phosphonates (where the ester group is replaced by an aldehyde) can be readily prepared [6], but using them directly in Wittig-Horner reactions is not practicable because of self-condensation. Reactions using aldehyde phosphonates protected as imines and acetals have been reported, but these reagents also have problems with side reactions, insufficient reactivity, and lability of products [7]. The silylaldimines are the functional equivalents of protected aldehyde phosphonates and have been used in Wittig-Horner-like reactions [8,9]; however, reaction conditions to form and use silylaldimines are not operationally convenient [9]. Solid base-promoted cross-aldol condensations have been successful, but only aromatic or heteroaromatic aldehydes were suitable as reactants [10]. Recently, acidic ionic liquid-catalyzed homologation of α,β-enals was reported, but the scope of the reaction did not include aliphatic aldehydes [11].We chose to further explore phosphonate chemistry with a better carbonyl protective group because the scope of the reaction would include a variety of aldehydes and ketones. Protection of carbonyl compounds as dimethylhydrazones is a common practice, and the incorporation and removal of this group have been studied extensively [12,13,14].

**Scheme 1:**
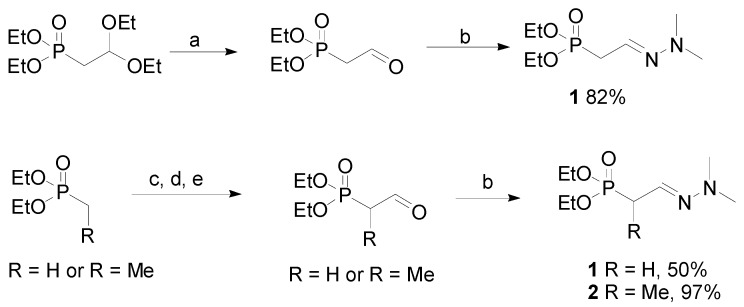
Preparation of phosphonate reagents **1** and **2**.

**Scheme 2:**
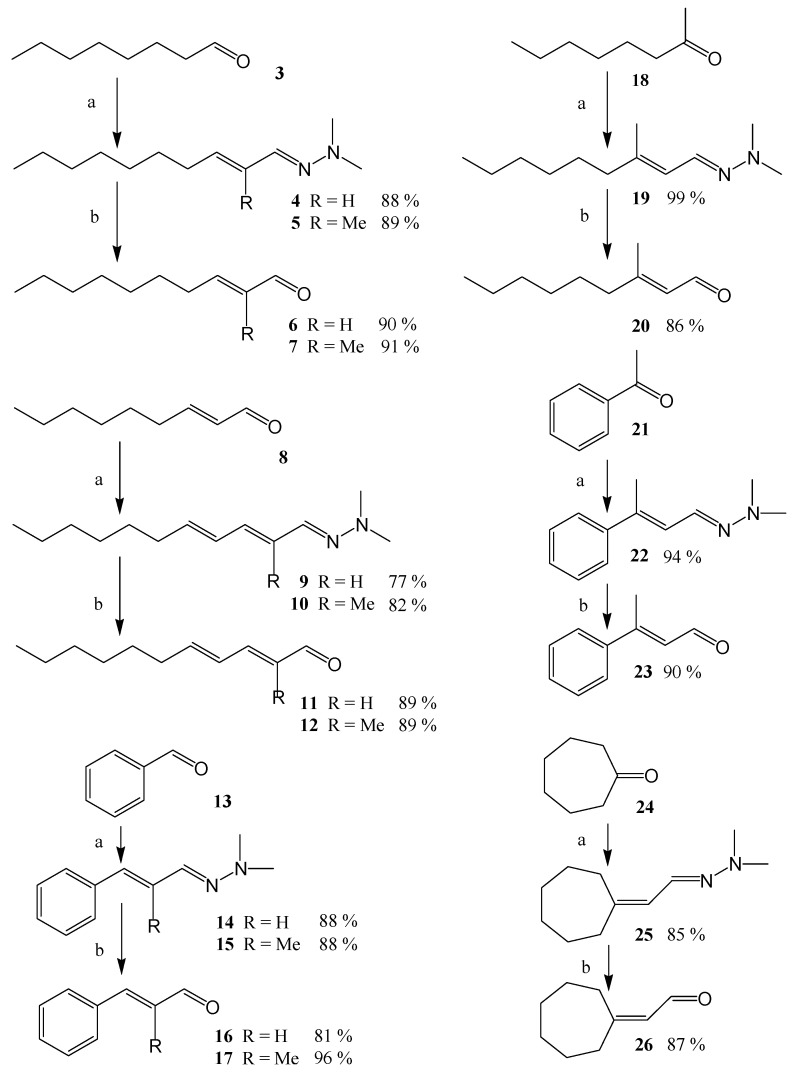
Reaction pathways and products.

This paper describes work detailing the preparation (Scheme 1) and use (Scheme 2) of diethyl methylformylphosphonate dimethylhydrazone (**1**) and diethyl 1-ethylformyl-2-phosphonate dimethyl-hydrazone (**2**) for the two-carbon homologation of a variety of aldehydes or ketones to α,β-unsaturated aldehydes after deprotection.

## 2. Results and Discussion

### 2.1. Preparation of Phosphonate Dimethylhydrazone Reagents

Reagent **1** could be prepared (Scheme 1) from commercially available diethyl-2,2-(diethoxy) ethylphosphonate by deprotection to give ethylformyl-2-phosphonate, then the aldehyde functional group could be protected as the dimethylhydrazone derivative [13]. Acetals of aldehydes have been deprotected by transfer to acetone, in the presence of certain catalysts [15,16,17,18]; therefore, deprotection of commercially available diethyl-2,2-(diethoxy)ethylphosphonate [7] would yield the required diethyl formylmethylphosphonate, a deprotection procedure, which needed only a small amount of water, would be preferable because the product, diethyl formylmethyl-2-phosphonate, is difficult to extract from water. Also, hydrolysis of the ethyl esters on phosphorous can occur in water [19].

The deprotection of diethyl-2,2-(diethoxy) ethylphosphonate was successfully carried out with a *p*-toluenesulfonic acid catalyst in acetone [18] containing only 1.5% water. The product contained 8% unused starting material, diethyl-2,2-(diethoxy)ethylphosphonate, but deprotection of the acetal with aqueous HCl also resulted in unreacted starting material [17].

The aldehyde functional group was then protected as the dimethylhydrazone derivative, with *N*,*N*-dimethylhydrazine, as done previously [19]. Phosphonate **1** was prepared in 82% overall yield from the starting material. The product still contained about 8% unused starting material, carried through the synthesis. The presence of starting material did not pose a problem when the phosphonate reagent was used for aldehyde homologation or ketone to aldehyde homologation.

Phosphonate reagent **1**, could also be prepared from commercially available diethyl methyl-phosphonate, by lithiation of diethyl methylphosphonate, formylation with dimethylformamide [19], followed by protection of the aldehyde function as the dimethylhydrazone derivative [13]. The overall yield for this synthetic pathway was only 50% (because of difficulty extracting water-soluble ethylformyl-2-phosphonate), so the other preparative route was preferred.

Phosphonate reagent **2** was prepared from diethyl ethylphosphonate by formylation, to prepare diethyl 1-propylformyl-2-phosphonate [19], and then the aldehyde functional group was protected as the dimethylhydrazone derivative. The overall yield was usually greater than 95%.

The new method requires the phosphonate reagents to be synthesized, whereas those for the original method are available commercially. Nevertheless, large batches of the new reagents can be prepared and stored, and these stocks could subsequently serve for syntheses of a variety of final products. Diethyl methylformylphosphonate can also be named (2-oxo-ethyl)-phosphonic acid diethyl ester and diethyl ethylformyl-2-phosphonate can also be named (1-methyl-2-oxo-ethyl)-phosphonic acid diethyl ester. Diethyl methylformylphosphonate dimethylhydrazone can also be named [2-(dimethyl-hydrazino)-ethyl]-phosphonic acid diethyl ester.and diethyl ethylformyl-2-phosphonate dimethylhydrazone can also be named [2-(dimethyl-hydrazino)-1-methyl-ethyl]-phosphonic acid diethyl ester. Alternative names for the aldehyde phosphonates and aldehyde phosphonate dimethylhydrazones **1** and **2** are given, but the simpler, and more intuitive, names were used in this paper.

### 2.2. Use of the Phosphonate 1 and 2 Reagents in Synthesis

Both **1** and **2** were readily converted to their corresponding anions with the base LDA and reacted smoothly with aldehydes or ketones, added in excess, to give unbranched- or methyl-branched, unsaturated aldehyde dimethylhydrazones with chains having two additional carbons (Scheme 2). Yields for the condensation step ranged from 77%–99%.

Deprotection of unsaturated aldehyde dimethylhydrazones was accomplished in 81%–96% yield, using a biphasic mixture of 1 M HCl and petroleum ether [14]. In the deprotection reaction, the dimethylhydrazone is initially converted to a water-soluble hydrochloride salt, and the free aldehyde then forms under the acidic conditions. Successful deprotection requires immediate migration of the liberated, but sometimes labile, aldehyde from the aqueous phase to the neutral petroleum ether phase. In the absence of petroleum ether, deprotection of unsaturated aldehyde dimethylhydrazones **9** and **10** gave a complex mixture of reaction products.

Using these reagents led to a two-step aldehyde homologation cycle (condensation and deprotection) instead of the previous three-step cycle. Overall yields, of unsaturated aldehyde products, for the two-step sequence ranged from 71% to 86%. Homologation of unbranched aldehyde starting materials with phosphonate **1** resulted in disubstituted α,β-unsaturated aldehyde products whereas homologation of unbranched aldehyde starting materials with phosphonate **2** resulted in trisubstituted α,β-unsaturated aldehyde products, with a methyl group at the 2-position. Homologation of methyl ketones with phosphonate **1** resulted in trisubstituted α,β-unsaturated aldehyde products, with a methyl group at the 3-position. The two-step homologation procedure gave a favorable (9.5:1 to 170:1) ratio of 2*E* to 2*Z* products from aldehyde starting materials and a ratio (2.0:1 to 2.2:1) of 2*E* to 2*Z* products from ketone starting materials. Homologation of cycloheptanone with phosphonate 2 resulted in an improved synthesis of cycloheptylidene-acetaldehyde **26**. The overall yield for the preparation of **26** increased from 42% [20] to 74%.

### 2.3. Assignment of Double-Bond Configuration

The major isomer configuration, at the α,β-double bond of disubstituted alkene compounds **4**, **6**, **9**, **11**, **14** and **16** (Scheme 2) was readily determined to be *E* by the coupling constant of *J*_2-3_ =15.1 to *J*_2-3_ = 16.1 Hz. The major isomer configuration of all trisubstituted double bonds, of compounds **5**, **7**, **10**, **12**, **15** and **17**, was determined by nuclear Overhauser enhancement (nOe) NMR methods. Irradiation at either H-1 or H-3 caused a nuclear Overhauser enhancement of 2.1% to 4.3% at H-1 or H-3 for compounds **5**, **7**, **10**, **12**, **15** and **17**, but only a nOe of 0% to 0.2% was observed for the methyl group protons for any of the compounds. Irradiation of the methyl group protons caused a nOe of just 0% to 0.5% for either H-1 or H-3. The data support the assignment of 2*E* configuration for compounds **5**, **7**, **10**, **12**, **15** and **17**, because the H-1 and H-3 protons are closer to each other than to the protons of the methyl group, attached to C-2.

The proportion of 2*Z*-isomer product was identified by its shorter GC retention time and nearly identical mass spectra, compared to the 2*E*-isomer [21], for compounds **19**, and **20**. The major isomer configuration of the trisubstituted double bonds, of compounds **19**, and **20** was determined to be *E* because of the later GC retention time, previously observed for (2*E*)-*N,N*-Dimethyl-*N’*-(dec-2-enylidene)-hydrazine and (2*E*)-2-Decenal [19]. Also, the reported proton NMR chemical shifts more closely matched the *E*-isomer of **20** reported previously [22]. We could not use nuclear Overhauser enhancement (nOe) NMR methods for additional verification because the proton chemical shifts for the methyl branch and protons on adjacent carbons were too close together for the both isomers.

The major isomer configuration of the trisubstituted double bonds, of compounds **22** and **23**, derived from methyl ketone starting materials, **6**, and **7** was determined by nuclear Overhauser enhancement (nOe) NMR methods. A moderate nOe enhancement was observed (2D NOESY experiments) between the methyl group protons and H-2, in the case of the minor isomer for both compounds. Only weak nOe enhancement was observed between the methyl group protons and H-2, in the case of the major isomer of compound **6** and no nOe enhancement was observed between the methyl group protons and H-2, in the case of the major isomer of compound **23**. The data support the assignment of 2*Z* configuration for the minor isomer of compounds **22** and **23** because the H-2 protons are closer to the protons of the methyl group, attached to C-3. Therefore, the major isomer has the *E*-configuration. The GC retention time for the major isomer was also later than that observed for the minor isomer, consistent with the assignment of *E*-configuration for the major isomer. Compound **23** has been prepared previously [23,24,25] and the chemical shift for the methyl branch carbon, attached to C-3, was δ 16.4 for the major *E*-isomer and δ 26.4 for the minor Z-isomer, consistent with reported ^13^C-NMR data [25].

## 3. Experimental

### General

Diethyl-2,2-(diethoxy)ethylphosphonate, diethyl methylphosphonate and diethylethylphosphonate were purchased from Alfa Aesar (Ward Hill, MA, USA). Diethyl ethylphosphonate can also be prepared in 98% yield by refluxing a mixture of triethyl phosphite and ethyl iodide for 3 h [26]. THF was purified by distillation from sodium benzophenone ketyl. Lithium diisopropylamide (LDA) was purchased from the Aldrich Chemical Co (Milwaukee, WI, USA) as a 2 M solution in a mixture of heptane/THF/ethylbenzene. Other organic reagents were obtained from Aldrich and were used without further purification. Non-aqueous reactions were performed under an atmosphere of dry argon in oven-dried glassware. Removal of solvent was accomplished by rotary evaporation at water aspirator pressure.

Progress of synthetic reactions was monitored by gas chromatography (GC). All reaction products were identified by GC/MS and the structures were additionally verified by NMR. Yields were corrected for purity. Both 2*E* and 2*Z* isomers were considered to be the product for all product compounds; however, the 2*E*:2*Z* isomer ratios are reported.

The Hewlett-Packard (HP) 5890 Series II gas chromatograph was equipped with flame ionization detector and split/splitless inlet and was interfaced to an HP ChemStation data system. The column was a DB-5 capillary (30 m × 0.25 mm, 0.25-μm film thickness, J&W Scientific, Folsom, CA, USA). Carrier gas was He. The oven temperature was programmed from 50 to 280 °C at 10 °C/min, and the detector temperature was 280 °C. The inlet temperature was 220 °C, and 1.0 µL sample injections were made in splitless mode. The inlet temperature was 120 °C, and 1.0 µL sample injections were made in splitless mode for analysis for compounds **9** and **10** because considerable degradation of unsaturated aldehyde dimethylhydrazones was observed during GC runs with higher inlet temperatures.

Electron impact mass spectra (70 eV) were obtained with an HP 5973 MSD instrument, interfaced to an HP 6890 GC, equipped with a splitless inlet. Several columns were used, but gave comparable results to that used for GC. The oven temperature was programmed from 50 to 250 °C at 10 °C/min; the inlet temperature was 220 °C and the transfer line temperature was 250 °C. The inlet temperature was 120 °C for compounds **9** and **10**.

^1^H-NMR, ^13^C-NMR and 2D NMR spectra were collected on a Bruker (Bellerica, MA, USA) Avance 500 spectrometer using a 5 mm broadband probe. Samples were dissolved in either CDCl_3_ or C_6_D_6_ (where indicated) and all spectra (^1^H and COSY at 500 MHz, ^13^C and DEPT at 125 Mhz) were acquired at 300° K. Trisubstituted double-bond “E” or “Z” assignments were based on 1D PFG-NOE experiments and 2D PFG-NOESY experiments. Chemical shifts are reported as parts per million from tetramethylsilane based on the lock solvent. Coupling constants (*J*) are in Hertz. Assignments were made with the help of NMR prediction software from Advanced Chemistry Development, Inc. (ACD/Labs) [27] and by analogy to known compounds. Although compounds **6**, **7**, **20**, **22**, **23** and **26** are known, spectral data are given because MS and NMR data are very difficult to find in the older chemical literature. Methyl branches are reported by the point of attachment to the carbon chain (e.g., CH_3_ at C-2).

*Diethyl methylformylphosphonate dimethylhydrazone* (**1**). Diethyl-2,2-(diethoxy)ethylphosphonate (5.31 g, 20.9 mmol) was added to acetone (200 mL), followed by water (3 mL) and *p*-toluenesulfonic acid (1.3 g, 6.8 mmol). The mixture was stirred at r.t., under an argon atmosphere for 4 days. A solution of NaHCO_3_ (570 mg, 6.8 mmol in 6 mL water) was added with stirring to quench the reaction and neutralize acid present. The pH was checked and found to be 7. After removal of solvent, aqueous oil remained. The oil was saturated by the addition of NaCl (25 g). The aqueous oil was repeatedly extracted with CH_2_Cl_2_ (100 mL then 2 × 50 mL), and the combined CH_2_Cl_2_ extracts were dried over anhydrous Na_2_SO_4_ and filtered. Solvent was removed to afford 5.59 g of crude diethyl methylformyl-2-phosphonate. Without further purification, the crude product was converted to the dimethylhydrazone (DMH) derivative by adding it to a mixture of CH_2_Cl_2_ (75 mL) containing anhydrous MgSO_4_ (5.3 g, 44 mmol) then *N*,*N*-dimethylhydrazine (1.76 g, 2.2 mL, 22 mmol) was added in one portion. The mixture was stirred (48 h) until analysis by GC showed complete protection of the aldehyde as the DMH derivative. Filtration, removal of solvent, and Kugelrohr distillation (oven temperature 62 °C, 0.05 Torr) afforded 4.41 g of oil containing **1** (purity 86%, yield corrected for purity 82%). The oil also contained 8% starting material, diethyl-2,2-(diethoxy)ethylphosphonate. MS of product: (EI) *m*/*z* (%) 222 (M^+^, 32), 180 (9), 152 (91), 125 (100), 122 (74), 108 (27), 97 (37), 85 (85), 71 (15), 58 (8), 44 (91). ^1^H-NMR δ 1.30 (6H, t, *J* = 7.0, C**H**_3_–CH_2_–O), 2.77 (6H, s, N–CH_3_), 2.81 (1H, dt, *J_1-2_* = 5.8, H-2), 4.09, (4H, q, *J* = 7.0, CH_3_–C**H**_2_–O), 6.47 (1H, br d, *J*_1-2_ = 5.8, H-1). ^13^C-NMR δ 16.4 (**C**H_3_–CH_2_–O), 31.3 (C-2), 42.9 (N–CH_3_), 61.9 (CH_3_–**C**H_2_–O), 126.1 (C-1). The MS and NMR spectral data were consistent with the reference spectral data from previous work [19].

*Alternative preparation of diethyl methylformylphosphonate dimethylhydrazone* (**1**). A solution of *n*-butyllithium (2.5 M, 25 mL, 0.213 mol, slight excess) was added to dry THF (80 mL) and the mixture was cooled in an argon atmosphere to dry ice-ethanol bath temperature (approx −78 °C). Diethyl methylphosphonate (9.13 g, 8.8 mL, 0.06 mol) was added dropwise and the mixture was stirred for an additional hr before dry dimethylformamide (DMF, distilled from CaH_2_, 4.8 mL, 0.066 mol) was added. After warming to 20 °C, ice cold 3 M aqueous HCl (300 mL) was added and the mixture was stirred 5 min. A fine white precipitate formed initially, and then the solution cleared and separated into two phases (liberation of the free phosphonate aldehyde). The phases were separated, and the organic phase was washed with 10 mL of alkaline brine solution (0.5 % NaHCO_3_ in saturated aqueous NaCl), dried over anhydrous MgSO_4_, and filtered. The acidic aqueous phase was repeatedly extracted with CH_2_Cl_2_ (5 × 50 mL), and the combined CH_2_Cl_2_ extracts were washed with alkaline brine solution (2 × 10 mL). The alkaline brine washes were combined and extracted again with CH_2_Cl_2_ (4 × 15 mL). All CH_2_Cl_2_ extracts were combined, dried over anhydrous MgSO_4_, and filtered. Solvent was removed from the dried organic phase and all dried CH_2_Cl_2_ extracts to afford 9.8 g of crude diethyl methylformyl-2-phosphonate, which still contained some DMF. Without further purification, the crude product was converted to the dimethylhydrazone (DMH) derivative by adding it to a mixture of CH_2_Cl_2_ (200 mL) containing anhydrous MgSO_4_ (14.4 g, 0.12 mol) then *N*,*N*-dimethylhydrazine (4.7 g, 6 mL, 0.08 mol) was added in one portion. The mixture was stirred (48 h) until analysis by GC showed complete protection of the aldehyde as the DMH derivative. Filtration, removal of solvent, and Kugelrohr distillation (oven temperature 44 °C, 0.02 Torr) afforded 7.1 g of **1** (purity 93%, yield corrected for purity 50%). MS (EI) *m*/*z* (%) 222 (M^+^, 32), 180 (9), 152 (91), 125 (100), 122 (74), 108 (27), 97 (37), 85 (85), 71 (15), 58 (8), 44 (91). EI-HRMS C_8_H_19_N_2_O_3_P calcd. for 222.1133 (obs. 222.1133). ^1^H-NMR δ 1.30 (6H, t, *J* = 7.0, C**H**_3_–CH_2_–O), 2.77 (6H, s, N–CH_3_), 2.81 (1H, dt, *J_1-2_* = 5.8, H-2), 4.09, (4H, q, *J* = 7.0, CH_3_–C**H**_2_–O), 6.47 (1H, br d, *J*_1-2_ = 5.8, H-1). ^13^C-NMR δ 16.4 (**C**H_3_–CH_2_–O), 31.3 (C-2), 42.9 (N–CH_3_), 61.9 (CH_3_–**C**H_2_–O), 126.1 (C-1).

*Diethyl ethylformyl-2-phosphonate dimethylhydrazone* (**2**). A solution of *n*-butyllithium (2.5 M, 85 mL, 0.213 mol, slight excess) was added to dry THF (250 mL) and the mixture was cooled in an argon atmosphere to dry ice-ethanol bath temperature (approx −78 °C). Diethyl ethylphosphonate (33.2 g, 32.4 mL, 0.20 mol) was added dropwise and the mixture was stirred for an additional hr before dry DMF (20 mL, 0.26 mol) was added. After warming to 20 °C, ice cold 3 M aqueous HCl (300 mL) was added and the mixture was stirred 5 min. A fine white precipitate formed initially, and then the solution cleared and separated into two phases (liberation of the free phosphonate aldehyde). The phases were separated, and the organic phase was washed with 30 mL of alkaline brine solution (0.5% NaHCO_3_ in saturated aqueous NaCl), dried over anhydrous MgSO_4_, and filtered. The acidic aqueous phase was repeatedly extracted with CH_2_Cl_2_ (5 × 150 mL), and the combined CH_2_Cl_2_ extracts were washed with 30 mL of alkaline brine solution. The alkaline brine washes were combined and extracted again with CH_2_Cl_2_ (4 × 15 mL). All CH_2_Cl_2_ extracts were combined, dried over anhydrous MgSO_4_, and filtered. Solvent was removed from the dried organic phase and all dried CH_2_Cl_2_ extracts to afford 44 g of crude diethyl ethylformyl-2-phosphonate, which still contained some DMF. Without further purification, the crude product was converted to the dimethylhydrazone (DMH) derivative by adding it to a mixture of CH_2_Cl_2_ (600 mL) containing anhydrous MgSO_4_ (48g, 0.4 mol) then *N*,*N*-dimethylhydrazine (15.6 g, 20 mL, 0.26 mol) was added in one portion. The mixture was stirred (48 h) until analysis by GC showed complete protection of the aldehyde as the DMH derivative. Filtration, removal of solvent, and Kugelrohr distillation (oven temperature 50 °C, 0.04 Torr) afforded 47.6 g of **3** (purity 98%, yield corrected for purity 97%). Isolated yields >95% were routinely obtained. MS (EI) *m*/*z* (%) 236 (M^+^, 34), 194 (8), 166 (82), 136 (42), 122 (10), 111 (12), 99 (100), 53 (12), 81 (12), 72 (11), 56 (12), 44 (36). EI-HRMS C_9_H_21_N_2_O_3_P calcd. for 236.1290 (obs. 236.1293) ^1^H-NMR δ 1.19 and 1.20 (6H, overlapping t, *J* = 7.0, C**H**_3_–CH_2_–O), 1.26 (3H, d, *J*_2-3_ = 7.3, H-3), 2.76 (1H, dt, *J_1-2_* = 4.1 and *J*_2-3_ = 7.3, H-2), 2.66 (6H, s, N–CH_3_), 3.99 and 4.00, (4H, overlapping q, *J* = 7.0, CH_3_–C**H**_2_–O), 6.38 (1H, br d, *J*_1-2_ = 4.1, H-1). ^13^C-NMR δ 12.7 (C-3), 16.4 (**C**H_3_–CH_2_–O), 36.0 and 37.1 (C-2, isomers), 43.1 (N–CH_3_), 62.0 (CH_3_–**C**H_2_–O), 132.6 (C-1).

*(2E)-N,N-Dimethyl-N’-(dec-2-enylidene)-hydrazine* (**4**). A commercial 2.0 M solution of LDA (5.2 mL, 10.4 mmol, slight excess) was added dropwise (reaction exothermic) to a 250 mL round-bottomed flask containing dry THF (25 mL), **1** (2.22 g, containing 2.06 g of **1**, 9.3 mmol) and a few mg of ethyl(triphenyl)phosphonium bromide, as an indicator, and the mixture was stirred in an argon atmosphere at r.t. for 1.5 h to ensure complete formation of the phosphonate ylide. A persistent red color developed in the solution when sufficient base was added to convert the phosphonate to its anion. Then, *n*-octanal (1.67 g, 13 mmol) was added dropwise; final color was yellow-orange. The mixture was stirred at r.t. for 5 h. Consumption of the phosphonate **1** was monitored by GC. Water (6 mL) was added to quench the reaction (excess water should be avoided because it leads to problematic emulsions during subsequent extraction steps). The solvent was removed by rotary evaporation to give an oily residue, which was extracted with ethyl acetate (5 × 25 mL). The combined extracts were washed with saturated aqueous NaCl solution (15 mL), dried over anhydrous Na_2_SO_4_ and filtered. Removal of solvent left a yellow oil. Kugelrohr distillation (oven temperature 59 °C, 0.05 Torr) afforded 1.80 g of **4** (89% purity by GC, yield corrected for purity 88%). By GC and GC-MS, the 2*E*/2*Z* isomer ratio was 8.5 to 1. MS (EI) *m*/*z* (%) 196 (M^+^, 72), 181 (8), 167 (2), 152 (19), 140 (7), 125 (14), 111 (100), 82 (40), 59 (21), 44 (32), 42 (19). EI-HRMS C_12_H_24_N_2_ calcd. for 196.1939 (obs. 196.1940). ^1^H-NMR δ 0.89 (3H, t, *J*_9-10_ = 6.9, H-10), 1.27-1.30 (8H, m, H-6 to H-9), 1.42 (2H, m, H-5), 2.15 (2H, m, *J*_3-4_ = 7.8, H-4), 2.84 (6H, s, N–CH_3_), 5.87 (1H, dd, *J*_3-4_ = 7.8 and *J*_2-3_ = 15.5, =CH, H-3), 6.20 (1H, dd, *J*_1-2_ = 9.1 and *J*_2-3_ = 15.5, =CH, H-2), 7.11 (1H, d, *J*_1-2_ = 9.1, =CH, H-1). ^13^C-NMR δ 13.3 (C-10), 22.6 (C-9), 29.0 (C-5, C-6 and C-7), 31.7 (C-8), 32.6 (C-4), 43.0 (N–CH_3_), 128.6 (C-2), 136.4 (C-3), 137.6 (C-1).

*(2E)-2-Decenal* (**6**). Deprotection was initiated by stirring **4** (1.10 g, containing 0.98 g **4**, 5.0 mmol) with 1 M HCl (50 mL) at room temperature for 5 min (**4** dissolves as the hydrochloride salt forms), and then petroleum ether (b.p. 35 °C to 60 °C, 50 mL) was added and stirring continued. After 4 h, the phases were separated and the aqueous phase was returned to the reaction vessel. Petroleum ether (30 mL) was added and the mixture was stirred at r.t. for an additional 1 h before the phases were separated. The combined petroleum ether phases were washed with alkaline brine solution (2 × 10 mL), and the brine phase was back-extracted once with petroleum ether (20 mL). The combined organic phases were dried, filtered, and removal of solvent afforded 0.75 g of the free aldehyde **6**. Purity by GC was 92%, and yield corrected for purity was 90%. The 2*E*:2*Z* isomer ratio was 25 to 1. The MS and NMR spectral data were consistent with the reference spectral data from previous work [9].

*(2E)-N,N-Dimethyl-N’-(2-methyl-dec-2-enylidene)-hydrazine* (**5**). Compound **5** was prepared in a way similar to compound **4**. The phosphonate anion was prepared from **2** (2.36 g, containing 2.31 g of **2**, 9.8 mmol) in THF (25 mL) by treatment with LDA (2.0 M, 5.2 mL, 10.4 mmol, slight excess), and *n*-octanal (1.92 g, 15 mmol) was subsequently added and the mixture was stirred at r.t. for 20 h. Water (3 mL) was added to quench the reaction and the reaction was worked up as before. Kugelrohr distillation (oven temperature 69 °C, 0.1 Torr) afforded 2.04 g of **5**. (90% purity by GC, yield corrected for purity 89%). By GC and GC-MS, the 2*E*/2*Z* isomer ratio was 8.2 to 1. MS (EI) *m*/*z* (%) 210 (M^+^, 68), 195 (24), 180 (3), 166 (82), 125 (100), 111 (13), 96 (29), 82 (37), 59 (18), 44 (31), 42 (15). EI-HRMS C_13_H_26_N_2_ calcd. for 210.2096 (obs. 210.2093). ^1^H-NMR δ 0.90 (3H, t, *J*_9-10_ = 7.0, H-10), 1.28-1.31 (8H, m, H-6 to H-9), 1.41 (2H, m, H-5), 1.84 (3H, s, CH_3_ attached to C-2), 2.20 (2H, m, H-4), 2.83 (6H, s, N–CH_3_), 5.61 (1H, dd, *J*_3-4_ = 6.9, =CH, H-3), 7.15 (1H, s, =CH, H-1). ^13^C-NMR δ 11.6 (CH_3_ attached to C-2), 14.1 (C-10), 22.6 (C-9), 28.1 (C-4), 29.3 (C-6 and C-7), 29.9 (C-5), 31.9 (C-8), 43.0 (N–CH_3_), 132.4 (C-2), 134.2 (C-3), 141.2 (C-1).

*(2E)-2-Methyl-2-Decenal* (**7**). Deprotection procedure was similar to that used for **6**. A solution containing **5** (1.15 g, containing 1.04 g **3c**, 5.0 mmol) and 1 M HCl (60 mL), to which petroleum ether (b.p. 35 °C to 60 °C, 60 mL) was added after 5 min, was stirred at room temperature for 6 h. The phases were separated and the aqueous phase was returned to the reaction vessel. Petroleum ether (40 mL) was added and the mixture was stirred at r.t. for an additional 1 h before the phases were separated. Workup of the reaction as for **6** resulted in 0.85 g of the free aldehyde **7** (purity by GC showed 90%, yield corrected for purity 91%). The 2*E*:2*Z* isomer ratio was 24 to 1. The MS and NMR spectral data were consistent with the reference spectral data from previous work [28].

*(2E,4E)-N’-(Undeca-2,4-dienylidene)-N,N-dimethyl-hydrazine* (**9**). Compound **9** was prepared in a way similar to compound **4**. The phosphonate anion was prepared from **1** (2.22 g, containing 2.06 g of **1**, 9.3 mmol) in THF (25 mL) by treatment with LDA (2.0 M, 5.2 mL, 10.4 mmol, slight excess), and (2*E*)-2-nonenal (1.68 g, 12 mmol) was subsequently added and the mixture was stirred at r.t. for 3 h. Water (6 mL) was added to quench the reaction and the reaction was worked up as before. Kugelrohr distillation (oven temperature 70 °C, 0.05 Torr) afforded 1.62 g of **9**. (92% purity by GC, yield corrected for purity 77%). By GC and GC-MS, the 2*E*/2*Z* isomer ratio was 16 to 1. MS (EI) *m*/*z* (%) 208 (M^+^, 18), 137 (3), 123 (100), 108 (2), 94 (8), 80 (11), 67 (5), 44 (7), 42 (3). EI-HRMS C_13_H_24_N_2_ calcd. for 208.1939 (obs. 208.1944). ^1^H-NMR δ 0.90 (3H, t, *J*_10-11_ = 7.2, H-11), 1.29–1.31 (6H, m, H-8 to H-10), 1.41 (2H, m, H-7), 2.12 (2H, m, *J*_5-6_ = 7.2, H-6), 2.87 (6H, s, N–CH_3_), 5.75, (1H, dt, *J*_5-6_ = 7.2 and *J*_4-5_ = 15.0, =CH, H-5), 6.13 (1H, dd, *J*_3-4_ = 10.0 and *J*_4-5_ = 15.0, =CH, H-4), 6.28 (1H, m, *J*_3-4_ = 10.0 and *J*_2-3_ = undeterminable in CDCl_3_, =CH, H-3), 6.28 (1H, m, *J*_1-2_ = 6.0 and *J*_2-3_ = undeterminable in CDCl_3_, =CH, H-2), 7.05 (1H, d, *J*_1-2_ = 6.0, =CH, H-1). ^1^H-NMR in C_6_D_6_ δ 0.98 (3H, t, *J*_10-11_ = 7.8, H-11), 1.29–1.31 (6H, m, H-8 to H-10), 1.41 (2H, m, H-7), 2.10 (2H, m, *J*_5-6_ = 7.2, H-6), 2.63 (6H, s, N–CH_3_), 5.77, (1H, dt, *J*_5-6_ = 7.2 and *J*_4-5_ = 15.1, =CH, H-5), 6.22 (1H, dd, *J*_3-4_ = 10.4 and *J*_4-5_ = 15.1, =CH, H-4), 6.41 (1H, dd, *J*_3-4_ = 10.4 and *J*_2-3_ = 15.1, =CH, H-3), 6.72 (1H, dd, *J*_1-2_ = 8.5 and *J*_2-3_ = 15.1, =CH, H-2), 7.14 (1H, d, *J*_1-2_ = 8.5, =CH, H-1). ^13^C-NMR δ 14.0 (C-11), 22.6 (C-10), 28.9 (C-8), 29.2 (C-7), 31.6 (C-9), 32.8 (C-6), 42.9 (N–CH_3_), 128.6 (C-2), 130.3 (C-4), 133.2 (C-3), 135.8 (C-5), 136.2 (C-1).

*(2E,4E)-2,4-undecadienal* (**11**). Deprotection procedure was similar to that used for **6**. A solution containing **9** (1.16 g, containing 1.07 g **9**, 5.1 mmol) and 1 M HCl (50 mL), to which petroleum ether (b.p. 35 °C to 60 °C, 50 mL) was added after 5 min, was stirred at room temperature for 24 h. The phases were separated and the aqueous phase was returned to the reaction vessel. Petroleum ether (40 mL) was added and the mixture was stirred at r.t. for an additional 1 h before the phases were separated. Workup of the reaction as for **6** resulted in 0.81 g of the free aldehyde **11** (purity by GC showed 93%, yield corrected for purity 89%). The 2*E*:2*Z* isomer ratio was 9.5 to 1. MS (EI) *m*/*z* (%) 166 (M^+^, 6), 137 92), 123 (4), 109 (5), 95 (12), 81 (100), 67 (15), 55 (11), 43 (10), 41 (18). ^1^H-NMR δ 0.91 (3H, t, *J*_10-11_ = 6.9, H-11), 1.30-1.32 (6H, m, H-8 to H-10), 1.47 (2H, m, *J*_6-7_ = 7.2, H-7), 2.24 (2H, dt, *J*_5-6_ = 6.6 and *J*_6-7_ = 7.2, H-6), 6.09 (1H, dd, *J*_1-2_ = 7.9 and *J*_2-3_ = 15.1, =CH, H-2), 5.79 (minor 2*Z* isomer, 1H, dd, *J*_1-2_ = 7.9 and *J*_2-3_ = 11.2, =CH, H-2), 6.31, (1H, m, *J*_5-6_ = 6.6 and *J*_4-5_ = undeterminable in CDCl_3_, =CH, H-5), 6.31 (1H, d, *J*_3-4_ = 9.8 and *J*_4-5_ = undeterminable in CDCl_3_, =CH, H-4), 7.10 (major 2*E* isomer: 1H, dd, *J*_3-4_ = 9.8 and *J*_2-3_ = 15.1, =CH, H-3), 6.93 (minor 2*Z* isomer, 1H, dd, *J*_3-4_ = 9.8 and *J*_2-3_ = 11.2, =CH, H-3), 9.55 (1H, d, *J*_1-2_ = 7.9, O=CH, H-1), 10.19 (minor 2*Z* isomer, 1H, d, *J*_1-2_ = 7.9, O=CH, H-1). The sample also contained about 6% of the 2*E*,4*Z* isomer: δ 6.16 (1H, dd, *J*_1-2_ = 7.9 and *J*_2-3_ = 15.4, =CH, H-2), 6.30 (1H, dd, *J*_4-5_ = 11.3, =CH, H-4), 7.45 1H, dd, *J*_3-4_ = 9.8 and *J*_2-3_ = 15.1, =CH, H-3), 9.63 (1H, d, *J*_1-2_ = 7.9, O=CH, H-1). ^1^H-NMR in C_6_D_6_ δ 0.99 (3H, t, *J*_10-11_ = 7.1, H-11), 1.27–1.32 (8H, m, H-7 to H-10), 1.91 (2H, m, *J*_5-6_ = 7.0, H-6), 5.79, (1H, dt, *J*_5-6_ = 7.0 and *J*_4-5_ = 15.1, =CH, H-5), 5.90 (1H, dd, *J*_3-4_ = 10.7 and *J*_4-5_ = 15.1, =CH, H-4), 6.03 (1H, dd, *J*_1-2_ = 7.8 and *J*_2-3_ = 15.4, =CH, H-2), 6.55 (1H, dd, *J*_3-4_ = 10.7 and *J*_2-3_ = 15.4, =CH, H-3), 9.54 (1H, d, *J*_1-2_ = 7.8, O=CH, H-1). ^13^C-NMR δ 14.0 (C-11), 22.5 (C-9), 28.5 (C-7), 28.8 (C-8), 31.6 (C-10), 33.2(C-6), 128.6 (C-4), 130.0 (C-2), 147.4 (C-5), 152.8 (C-3), 193.9 (C-1).

*(2E, 4E)-N’-(2-Methyl-undeca-2,4-dienylidene)-N,N-dimethyl-hydrazine* (**10**). Compound **10** was prepared in a way similar to compound **4**. The phosphonate anion was prepared from **2** (2.36 g, containing 2.31 g of **2**, 9.8 mmol) in THF (25 mL) by treatment with LDA (2.0 M, 5.2 mL, 10.4 mmol, slight excess), and (2*E*)-2-nonenal (1.68 g, 12 mmol) was subsequently added and the mixture was stirred at r.t. for 20 h. Water (3 mL) was added to quench the reaction and the reaction was worked up as before. Kugelrohr distillation (oven temperature 78 °C, 0.06 Torr) afforded 2.09 g of **10**. (85% purity by GC, yield corrected for purity 82%). By GC and GC-MS, the 2*E*/2*Z* isomer ratio was 39 to 1. MS (EI) *m*/*z* (%) 222 (M^+^, 14), 137 (100), 108 (7), 94 (13), 44 (4), 43 (5). EI-HRMS C_14_H_26_N_2_ calcd. for 222.2096 (obs. 222.2102). ^1^H-NMR δ 0.91 (3H, t, *J*_10-11_ = 7.0, H-11), 1.29-1.31 (6H, m, H-8 to H-10), 1.43 (2H, m, H-7), 1.96 (CH_3_ attached to C-2), 2.16 (2H, m, *J*_5-6_ = 7.2, H-6), 2.87 (6H, s, N–CH_3_), 5.77, (1H, dt, *J*_5-6_ = 7.2 and *J*_4-5_ = 15.1, =CH, H-5), 6.12 (1H, d, *J*_3-4_ = 11.2, =CH, H-3), 6.44 (1H, dd, *J*_3-4_ = 11.2 and *J*_4-5_ = 15.1, =CH, H-4), 7.08 (1H, s, =CH, H-1). ^13^C-NMR δ 11.8 (CH_3_ attached to C-2), 14.1 (C-11), 22.6 (C-10), 28.9 (C-8), 29.4 (C-7), 31.7 (C-9), 33.2 (C-6), 43.2 (N–CH_3_), 126.7 (C-4), 131.2 (C-2), 133.2 (C-3), 135.8 (C-5), 140.0 (C-1).

*(2E, 4E)-2-Methyl-2,4-undecadienal* (**12**). Deprotection procedure was similar to that used for **6**. A solution containing **10** (1.29 g, containing 1.10 g **10**, 5.0 mmol) and 1 M HCl (50 mL), to which petroleum ether (b.p. 35 °C to 60 °C, 50 mL) was added after 5 min, was stirred at room temperature for 48 h. The phases were separated and the aqueous phase was returned to the reaction vessel. Petroleum ether (40 mL) was added and the mixture was stirred at rt for an additional 1 h before the phases were separated. Workup of the reaction as for **6** resulted in 0.91 g of the free aldehyde **12** (purity by GC showed 88%, yield corrected for purity 89%). The 2*E*:2*Z* isomer ratio was 30 to 1. MS (EI) *m*/*z* (%) 180 (M^+^, 11), 109 (6), 95 (100), 81 (10), 79 (9), 67 (12), 55 (5), 53 (5), 43 (5), 41 (10). ^1^H-NMR δ 0.91 (3H, t, *J*_10-11_ = 6.8, H-11), 1.32–1.34 (6H, m, H-8 to H-10), 1.48 (2H, m, H-7), 1.85 (CH_3_ attached to C-2), 2.26 (2H, dt, *J*_5-6_ = 7.2, H-6), 6.26, (1H, dt, *J*_4-5_ = 15.0 and *J*_5-6_ = 7.2, =CH, H-5), 6.54 (1H, dd, *J*_3-4_ = 11.2 and *J*_4-5_ = 15.0, =CH, H-4), 6.84 (1H, d, *J*_3-4_ = 11.2, =CH, H-3), 9.44 (major 2*E* isomer, 1H, s, O=CH, H-1), 10.28 (minor 2*Z* isomer, 1H, s, O=CH, H-1), The sample also contained a trace amount of the 2*E*,4*Z* isomer: δ 6.13 (1H, d, *J*_3-4_ = 11.2, =CH, H-3), 6.85 (1H, dd, *J*_3-4_ = 11.2 and *J*_4-5_ = 11.3, =CH, H-4), 9.51 (1H, s, O=CH, H-1). ^13^C-NMR δ 14.0 (C-11), 22.5 (C-9), 28.5 (C-7), 28.8 (C-8), 31.6 (C-10), 33.2(C-6), 128.6 (C-4), 130.0 (C-2), 147.4 (C-4), 152.8 (C-3), 193.9 (C-1).

*(2E)-N,N-Dimethyl-N’-(3-phenyl-allylidene)-hydrazine* (**14**). Compound **14** was prepared in a way similar to compound **4**. The phosphonate anion was prepared from **1** (2.22 g, containing 2.06 g of **1**, 9.3 mmol) in THF (25 mL) by treatment with LDA (2.0 M, 5.2 mL, 10.4 mmol, slight excess), and benzaldehyde (1.11 g, 10.5 mmol) was subsequently added and the mixture was stirred at r.t. for 5 h. Water (3 mL) was added to quench the reaction and the reaction was worked up as before. Kugelrohr distillation (oven temperature 61 °C, 0.1 Torr) afforded 1.62 g of **14**. (88% purity by GC, yield corrected for purity 88%). By GC and GC-MS, the 2*E*/2*Z* isomer ratio was 50 to 1. The MS and NMR spectral data were consistent with the reference spectral data from previous work [13].

*(2E)-Cinnamaldehyde* (**16**). Deprotection procedure was similar to that used for **6**. A solution containing **14** (0.98 g, containing 0.86 g **14**, 4.9 mmol) and 1 M HCl (50 mL), to which petroleum ether (b.p. 35 °C to 60 °C, 50 mL) was added after 5 min, was stirred at room temperature for 18 h. The phases were separated and the aqueous phase was returned to the reaction vessel. Petroleum ether (50 mL) was added and the mixture was stirred at r.t. for an additional 1 h before the phases were separated. Workup of the reaction as for **6** resulted in 0.61 g of the free aldehyde **16** (purity by GC showed 86%, yield corrected for purity 81%). The 2*E*:2*Z* isomer ratio was 170 to 1. The MS and NMR spectral data were consistent with the reference spectral data from previous work [9].

*(2E)-N,N-Dimethyl-N’-(2-methyl-3-phenyl-allylidene)-hydrazine* (**15**). Compound **15** was prepared in a way similar to compound **4**. The phosphonate anion was prepared from **2** (2.36 g, containing 2.31 g of **2**, 9.8 mmol) in THF (25 mL) by treatment with LDA (2.0 M, 5.2 mL, 10.4 mmol, slight excess), and benzaldehyde (1.11 g, 10.5 mmol) was subsequently added and the mixture was stirred at r.t. for 20 h. Water (3 mL) was added to quench the reaction and the reaction was worked up as before. Kugelrohr distillation (oven temperature 69 °C, 0.1 Torr) afforded 1.82 g of **15**. (89% purity by GC, yield corrected for purity 88%). By GC and GC-MS, the 2*E*/2*Z* isomer ratio was 25 to 1. The MS and NMR spectral data were consistent with the reference spectral data from previous work [13].

*(2E)-Methyl cinnamaldehyde* (**17**). Deprotection procedure was similar to that used for **6**. A solution containing **15** (1.12 g, containing 1.00 g **15**, 5.3 mmol) and 1 M HCl (50 mL), to which petroleum ether (b.p. 35 °C to 60 °C, 50 mL) was added after 5 min, was stirred at r.t. for 48 h. The phases were separated and the aqueous phase was returned to the reaction vessel. Petroleum ether (40 mL) was added and the mixture was stirred at r.t. for an additional 1 hr before the phases were separated. Workup of the reaction as for **6** resulted in 0.78 g of the free aldehyde **17** (purity by GC showed 96%, yield corrected for purity 96%). The 2*E*:2*Z* isomer ratio was 56 to 1. The MS and NMR spectral data were consistent with the reference spectral data from previous work [9].

*N,N-Dimethyl-N'-(3-methyl-non-2-enylidene)-hydrazine* (**19**). A commercial 2.0 M solution of LDA, (4.4 mL, 8.8 mmol, slight excess) was added dropwise (reaction exothermic) to a 250 mL round-bottomed flask containing dry THF (24 mL), **1** (1.78 g, 8 mmol) and a few mg of ethyl(triphenyl)phosphonium bromide, as an indicator, and the mixture was stirred in an argon atmosphere at r.t. for 1.5 h to ensure complete formation of the phosphonate ylide. A persistent red color developed in the solution when sufficient base was added to convert the phosphonate to its anion. Then, 2-octanone (**18**, 1.44 g, 11.2 mmol) was added dropwise; final color was yellow-orange. The mixture was stirred at r.t. for 22 h. Consumption of the phosphonate **1** was monitored by GC. Water (6 mL) was added to quench the reaction (excess water should be avoided because it leads to problematic emulsions during subsequent extraction steps). The solvent was removed by rotary evaporation to give an oily residue, which was extracted with ethyl acetate (5 × 25 mL). The combined extracts were washed with saturated aqueous NaCl solution (15 mL), dried over anhydrous Na_2_SO_4_ and filtered. Yellow oil remained after removal of solvent. Kugelrohr distillation (oven temperature 60 °C, 0.10 Torr) afforded 1.95 g of **19** (82% purity by GC, yield corrected for purity >99%). By GC and GC-MS, the 2*E*/2*Z* isomer ratio was 2.2 to 1. MS (EI) *m*/*z* (%) 197, (M^+^ 1, 197, 14), 196 (M^+^, 100), 181 (27), 166 (7), 154 (17), 139 (13), 125 (58), 110 (21), 96, (75), 94 ((48), 82 (36), 67 (18), 59 (38), 44 (41). ^1^H-NMR, major 2*E*-isomer, δ 0.89 (3H, t, *J*_8-9_ = 6.8, H-9), 1.29 (6H, *m*, H-6, H-7, and H-8) 1.45 (2H, m, H-5), 1.83 (3H, s, H-10 which is the CH_3_ attached to C-3), 2.09 (2H, t, *J*_4-5_ = 7.6, H-4), 2.85 (6H, s, N–CH_3_, H-11), 5.99 (1H, d, *J*_1-2_ = 9.3, H-2), 7.26 (1H, br, H-1). ^13^C-NMR δ 14.1 (C-9), 17.0 (C-10), 22.6 (C-3), 27.6 (C-5), 28.9 (C-6), 31.7 (C-7), 40.0 (C-4), 43.3 (C-11), 122.5 (C-2), 128.2 (C-1), 141.3 (C-3). ^1^H-NMR, minor 2Z-isomer, δ 0.89 (3H, t, *J*_8-9_ = 6.8, H-9), 1.29 (6H, m, H-6, H-7, and H-8) 1.45 (2H, m, H-5), 1.81 (3H, s, H-10 which is the CH_3_ attached to C-3), 2.22 (2H, t, *J*_4-5_ = 7.5, H-4), 2.84 (6H, s, N-CH_3_, H-11), 5.99 (1H, d, *J*_1-2_ = 9.3, H-2), 7.16 (1H, br, H-1). ^13^C-NMR δ 14.1 (C-9), 22.6 (C-8), 24.0 (C-10), 28.0 (C-5), 29.1 (C-6), 31.7 (C-7), 32.5 (C-4), 43.3 (C-11), 123.5 (C-2), 128.4 (C-1), 135.2 (C-3).

*3-Methyl-non-2-enal* (**20**). Deprotection was initiated by stirring **19** (1.80 g, containing 1.48 g **19**, 7.5 mmol) with 1 M HCl (100 mL) at room temperature for 5 min (**19** dissolves as the hydrochloride salt forms), and then hexane (100 mL) was added and stirring continued. After 3 h, the phases were separated and the aqueous phase was returned to the reaction vessel. Hexane (100 mL) was added and the mixture was stirred at r.t. for an additional 1 h before the phases were separated. The combined hexane phases were washed with alkaline brine solution (2 × 10 mL), and the brine phase was back-extracted once with hexane (20 mL). The combined organic phases were dried, filtered, and removal of solvent afforded 1.04 g of the free aldehyde **20**. Purity by GC was 95%, and yield corrected for purity was 86%. The 2*E*:2*Z* isomer ratio was 2.0 to 1. MS (EI) *m*/*z* (%) 154 (M^+^, 5), 139 (17), 125 (11), 111 (5), 97, (100), 84 (61), 69 (22), 55 (23), 41 (36). ^1^H-NMR, major 2*E*-isomer, δ 0.89 (3H, t, *J*_8-9_ = 6.6, H-9), 1.29 (2H, m, H-7), 1.30 (4H, m, H-6 and H-8), 1.50 (2H, m, H-5), 2.16 (3H, s, H-10 which is the CH_3_ attached to C-3), 2.21 (2H, t, *J*_4-5_ = 7.5, H-4), 5.88 (1H, d, *J*_1-2_ = 8.2, H-2), 9.99 (1H, d, *J*_1-2_ = 8.2, H-1). ^13^C-NMR δ 14.1 (C-9), 17.5 (C-10), 22.5 (C-8), 27.1 (C-5), 28.8 (C-6), 31.5 (C-7), 40.6 (C-4), 127.3 (C-2), 164.4 (C-3), 191.1 (C-1). ^1^H-NMR, minor 2*Z*-isomer, δ 0.89 (3H, t, *J*_8-9_ = 6.6, H-9), 1.29 (2H, m, H-7), 1.30 (2H, m, H-8), 1.32 (2H, m, H-6), 1.54 (2H, m, H-5), 1.97 (3H, s, H-10 which is the CH_3_ attached to C-3), 2.57 (2H, t, *J*_4-5_ = 7.6, H-4), 5.87 (1H, d, *J*_1-2_ = 8.2, H-2), 9.96 (1H, d, *J*_1-2_ = 8.2, H-1). ^13^C-NMR δ 14.0 (C-9), 22.5 (C-8), 25.0 (C-10), 28.8 (C-5), 29.0 (C-6), 31.5 (C-7), 32.6 (C-4), 128.4 (C-2), 164.9 (C-3), 190.7 (C-1). The MS and NMR spectral data were consistent with the reference spectral data from previous work [22]. 

*N,N-Dimethyl-N'-(3-phenyl-but-2-enylidene)-hydrazine* (**22**). Compound **22** was prepared in a way similar to compound **19**. The phosphonate anion was prepared from **1** (1.78 g, 8 mmol) in THF (25 mL) by treatment with LDA (2.0 M, 4.4 mL, 8.8 mmol, slight excess), and acetophenone (**21**, 1.35 g, 11.2 mmol) was subsequently added and the mixture was stirred at r.t. for 22 h. Water (6 mL) was added to quench the reaction and the reaction was worked up as before. Kugelrohr distillation (oven temperature 69 °C, 0.1 Torr) afforded 1.64 g of **22**. (86 % purity by GC, yield corrected for purity 94%). By GC and GC-MS, the 2*E*/2*Z* isomer ratio was 2.1 to 1. MS (EI) *m*/*z* (%) 189 (M^+^ 1, 14), 188 (M^+^, 100), 173 (15), 158 (15), 144 (84), 128 (22), 115 (61), 103 (9), 91 (18), 77 (15), 58 (15), 42 (13). ^1^H-NMR, major 2*E*-isomer, δ 2.25 (3H, d, *J*_2-10_ = 1.2, H-10 which is the CH_3_ attached to C-3), 2.85 (6H, s, N-CH_3_, H-11), 6.68 (1H, dq, *J*_1-2_ = 9.3 and *J*_2-10_ = 1.1, H-2), 7.12 (1H, d, *J*_1-2_ = 9.3, H-1), 7.26 (1H, m, H-7), 7.35 (2H, t, *J*_5-6_ = 8.0, H-6), 7.51 (2H, d, *J*_5-6_ = 8.1, H-5). ^13^C-NMR δ 16.0 (C-10), 43.0 (C-11), 125.0 (C-2), 125.4 (C-5), 127.1 (C-7), 128.3 (C-6), 135.6 (C-1), 139.4 (C-4), 142.6 (C-3). ^1^H-NMR, minor 2*Z*-isomer, δ 2.18 (3H, d, *J*_2-10_ = 1.3, H-10 which is the CH_3_ attached to C-3), 2.78 (6H, s, N–CH_3_, H-11), 6.30 (1H, dq, *J*_1-2_ = 9.4 and *J*_2-10_ = 1.3, H-2), 7.26 (1H, m, H-7), 7.35 (2H, t, *J*_5-6_ = 8.0, H-6), 7.41 (1H, d, *J*_1-2_ = 9.3, H-1), 7.51 (2H, d, *J*_5-6_ = 8.1, H-5). ^13^C-NMR δ 25.6 (C-10), 43.0 (C-11), 125.4 (C-2 and C-5), 127.2 (C-7), 128.3 (C-6), 133.3 (C-1), 139.4 (C-4), 141.4 (C-3). This compound has been prepared previously [23].

*3-Phenyl-but-2-enal* (**23**). The deprotection procedure was similar to that used for **19**. A solution containing **22** (1.49 g, containing 1.28 g **22**, 6.8 mmol) and 1 M HCl (100 mL), to which hexane (100 mL) was added after 5 min, was stirred at room temperature for 3 h. The phases were separated and the aqueous phase was returned to the reaction vessel. Hexane (100 mL) was added and the mixture was stirred at r.t. for an additional 1 h before the phases were separated. Workup of the reaction as for **19** resulted in 0.95 g of the free aldehyde **23** (purity by GC showed 93%, yield corrected for purity 90%). The 2*E*:2*Z* isomer ratio was 2.1 to 1. MS (EI) *m*/*z* (%) 146 (M^+^, 46), 145 (100), 131 (24), 115 (42), 103 (15), 91 (17), 77 (11), 63 (6), 51 (10). ^1^H-NMR, major 2*E*-isomer, δ 2.59 (3H, s, H-10 which is the CH_3_ attached to C-3), 6.42 (1H, d, *J*_1-2_ = 7.7, H-2), 7.42 (1H, m, H-7), 7.43 (1H, m, H-6), 7.43 (1H, dd, *J*_5-6_ = 6.0 and *J*_5-7_ = 2.5, H-5), 10.20 (1H, d, *J*_1-2_ = 7.9, H-1). ^13^C-NMR δ 16.4 (CH_3_ attached to C-3), 126.3 (C-5) 127.3 (C-2), 128.8 (C-6), 130.1 (C-7), 140.6 (C-4), 157.6 (C-3), 191.1 (C-1), ^1^H-NMR, minor 2*Z*-isomer, δ 2.33 (3H, s, H-10 which is the CH_3_ attached to C-3), 6.15 (1H, d, *J*_1-2_ = 8.1, H-2), 7.31 (1H, dd, *J*_5-6_ = 7.1 and *J*_5-7_ = 3.3, H-5), 7.42 (2H, m, H-6 and H-7), 9.49 (1H, d, *J*_1-2_ = 8.2, H-1). ^13^C-NMR δ 26.4 (CH_3_ attached to C-3), 128.4 (C-5 and C-6), 129.2 (C-2 and C-7), 138.4 (C-4), 162.1 (C-3), 193.4 (C-1). The MS and NMR spectral data were consistent with the reference spectral data from previous work [24,25].

*N'-(2-Cycloheptylidene-ethylidene)-N,N-dimethyl-hydrazine* (**25**). Compound **25** was prepared in a way similar to compound **19**. The phosphonate anion was prepared from **1** (1.78 g, 8 mmol) in THF (25 mL) by treatment with LDA (2.0 M, 4.4 mL, 8.8 mmol, slight excess), cycloheptanone (**24**, 1.68 g, 11.2 mmol) was subsequently added and the mixture was stirred at r.t. for 22 h. Water (6 mL) was added to quench the reaction and the reaction was worked up as before. Kugelrohr distillation (oven temperature 61 °C, 0.1 Torr) afforded 1.40 g of **25**. (88% purity by GC, yield corrected for purity 85%) MS (EI) *m*/*z* (%) 181 (M^+^ 1, 13), 180 (M^+^, 100), 165 (64), 138 (38), 136 (37), 123 (22), 112 (41), 94 (41), 80 (50), 67 (26), 59 (26), 44 (30). ^1^H-NMR δ 1.52 (2H, m, H-6), 1.54 (2H, m, H-7), 1.62 (2H, m, H-5), 1.64 (2H,m , H-8), 2.32 (2H, t, *J*_4-5_ = 5.8, H-4), 2.46 (2H, td, *J*_8-9_ = 6.1 and *J*_2-9_ = 1.0, H-9), 2.85 (6H, s, N-CH_3_, H-10), 5.98 (1H, dp, *J*_1-2_ = 6.0 and *J*_2-9_ = 1.0, H-2), 7.30 (1H, br, H-1). ^13^C-NMR δ 27.1 (C-8), 28.8 (C-5), 29.0 (C-6), 29.7 (C-7), 30.6 (C-9), 43.2 (C-10), 123.1 (C-2), 128.2 (C-1), 146.9 (C-3).

*Cycloheptylidene-acetaldehyde* (**26**). The deprotection procedure was similar to that used for **19**. A solution containing **25** (1.27 g, containing 1.11 g **25**, 5.1 mmol) and 1 M HCl (100 mL), to which hexane (100 mL) was added after 5 min, was stirred at room temperature for 3 h. The phases were separated and the aqueous phase was returned to the reaction vessel. Hexane (100 mL) was added and the mixture was stirred at r.t. for an additional 1 h before the phases were separated. Workup of the reaction as for **19** resulted in 0.76 g of the free aldehyde **26** (purity by GC showed 98%, yield corrected for purity 87%). MS (EI) *m*/*z* (%) 138 (M^+^, 41), 137 (12), 123 (18), 110 (48), 109 (51), 95, (100), 81 (66), 67 (66), 55 (35), 41 (46).. ^1^H-NMR δ 1.55 (2H, m, H-6), 1.57 (2H, m, H-7), 1.69 (2H, m, H-5), 1.73 (2H,m , H-8), 2.45 (2H, t, *J*_4-5_ = 6.0, H-4), 2.84 (2H, t, *J*_8,9_ = 6.0, H-8), 5.85 (1H, d, *J*_1-2_ = 8.1, H-2), 9.9 (1H, d, *J*_1-2_ = 8.1, H-1). ^13^C-NMR δ 27.4 (C-5, C-8), 28.6 (C-6), 29.3 (C-7), 30.5 (C-9), 38.9 (C-4), 127.6 (C-2), 170.2 (C-3), 191.1 (C-1), The MS and NMR spectral data were consistent with the reference spectral data from previous work [20].

## 4. Conclusions

The new phosphonate reagents **1** and **2** were prepared without difficulty from sufficiently inexpensive starting materials and were stable enough to be distilled. The starting materials are thus inexpensive and the homologation reactions are sufficiently robust, so use of this two-step process can be commercially viable. Subsequent removal of the dimethylhydrazone protective group could be easily accomplished, even for delicate product aldehydes. Furthermore, the new method eliminates the use and disposal of some metal-containing reagents (powerful reducing agents such as lithium aluminum hydride or diisobutylaluminum hydride or oxidants such as pyridinium dichromate or manganese dioxide). This more concise homologation chemistry could be applied to the practical synthesis of a variety of natural products, pharmaceuticals, and other compounds. This new protocol should replace the usual three-step Horner-Wadsworth-Emmons [1] aldehyde or ketone homologation scheme.

## Figures and Tables

*Reagents and conditions*: Preparation of phosphonate **1**: (a) diethyl-2,2-(diethoxy)ethylphosphonate, acetone, 1.5% water, *p*-toluenesulfonic acid, 25 °C, 96 h; (b) H_2_N-NMe_2_, MgSO_4_, CH_2_Cl_2_, 25 °C, 48 h. Alternative preparation of **1**: (c) diethyl methylphosphonate, THF, BuLi; (d) Me_2_NCHO; (e) 3 M HCl, 1a–1c in one pot; (b) H_2_N-NMe_2_, MgSO_4_, CH_2_Cl_2_, 25 °C, 48 h. Preparation of phosphonate **2**: (c) diethyl ethylphosphonate, THF, BuLi; (d) Me_2_NCHO; (e) 3 M HCl, 1a–1c in one pot; (b) H_2_N-NMe_2_, MgSO_4_, CH_2_Cl_2_, 25 °C, 48 h.

*Reagents and conditions*: (a) Phosphonate **1** or **2**, LiN(*i*Pr)_2_, THF, 25 °C, 3 to 20 h, time varies with compound; (b) 1 M HCl and Pet ether, 25 °C, 5 to 48 h, time varies with compound.
